# Estimating LOCP cancer mortality rates in small domains in Spain using its relationship with lung cancer

**DOI:** 10.1038/s41598-021-01765-7

**Published:** 2021-11-15

**Authors:** Garazi Retegui, Jaione Etxeberria, María Dolores Ugarte

**Affiliations:** 1 Statistics, Computer Science and Mathematics, Public University of Navarre, 31006 Pamplona, Spain; 2 Institute for Advanced Materials and Mathematics (INAMAT2), Public University of Navarre, 31006 Pamplona, Spain; 3 Institute of Health Research (IdiSNA), 31008 Pamplona, Spain

**Keywords:** Cancer, Risk factors

## Abstract

The distribution of lip, oral cavity, and pharynx (LOCP) cancer mortality rates in small domains (defined as the combination of province, age group, and gender) remains unknown in Spain. As many of the LOCP risk factors are preventable, specific prevention programmes could be implemented but this requires a clear specification of the target population. This paper provides an in-depth description of LOCP mortality rates by province, age group and gender, giving a complete overview of the disease. This study also presents a methodological challenge. As the number of LOCP cancer cases in small domains (province, age groups and gender) is scarce, univariate spatial models do not provide reliable results or are even impossible to fit. In view of the close link between LOCP and lung cancer, we consider analyzing them jointly by using shared component models. These models allow information-borrowing among diseases, ultimately providing the analysis of cancer sites with few cases at a very disaggregated level. Results show that males have higher mortality rates than females and these rates increase with age. Regions located in the north of Spain show the highest LOCP cancer mortality rates.

## Introduction

According to the last GLOBOCAN estimates^1^, lip, oral cavity and pharynx cancers combined (LOCP onward) are responsible for 710,237 incident cases (period 2008–2012) and 358,536 deaths worldwide (period 1989–2017), accounting for about 3.9% of all cancer cases and 3.8% of cancer deaths. In Europe, LOCP cancer caused 53,200 deaths representing 2.8% of all cancer deaths^2^. Large differences have been observed for these cancers among countries. In this study, we will focus on the particular case of Spain. More precisely, our interest lies in looking into geographical LOCP cancer mortality patterns in detail. It is already known that LOCP cancer mortality mainly affects males and is considerably less lethal in women^3,4^. Another important factor in this cancer is age. Generally, rates increase with age^3,5^ but some differences are found by gender. Therefore, it is particularly interesting to analyze possible differences of geographical LOCP cancer mortality patterns by gender and age groups. Moreover, Spain is divided into autonomous regions, each one including one or more provinces (see Supplementary Information 1 online for more detail). Urbanization and industrialization have not developed in the same way in all provinces. Spain is also an heterogeneous country regarding lifestyle, socioeconomic factors, and health services. All these aspects could lead different geographical patterns for each cancer location^6–8^. Different studies for Spain and other countries show gender-specific LOCP cancer mortality patterns but age-specific patterns were not considered^9^. Aggregating counts over all age groups inside a region is a common practice for studying and modeling age-standardized mortality rates. However, in this work we are interested in providing age- and gender-specific rates in each region^10^ to give an in-depth description of the spatial distribution of LOCP mortality rates. However, when LOCP cancer mortality cases are analyzed by a combination of region, age, and gender (small domains), counts are scarce and it becomes difficult to fit univariate spatial models including all effects and their possible interactions. Therefore, alternative multivariate approaches are needed. One of such approach when the diseases are known to share common risk factors is the use of shared component models. These models belong to the class of models described by Held et al.^11^ and they assume dependence among diseases a priori. For example, Held et al.^12^ used them to jointly analyze four types of gastrointestinal infectious diseases. Shared component models are a powerful approach to borrow strength among diseases taking into account the possible correlation between the corresponding spatial patterns together with the possible specificity of each cancer site. Due to the inherent relationships between LOCP and lung cancers and tobacco consumption, LOCP and lung cancer mortality cases are jointly analyzed in this paper using different age- and gender-specific shared component models.

## LOCP and lung cancer mortality data

This study is based on LOCP and lung cancer mortality cases, and the population at risk reported by the Spanish Statistical Office (INE) in the period 2011–2015. LOCP cancer corresponds to codes C00-C14 and lung cancer corresponds to codes C33–34 of the International Classification of Diseases-10. The number of deaths were divided by 5-year age groups and gender in the 47 provinces of continental Spain shown in Supplementary Information 1. A total of 10,736 LOCP cancer deaths were registered, 8047 (75%) were males and 2689 (25%) females. In the case of lung cancer 100,265 deaths were registered; 81,910 (82%) of these deaths were males and 18,355 (18%) females.

**Figure 1:**
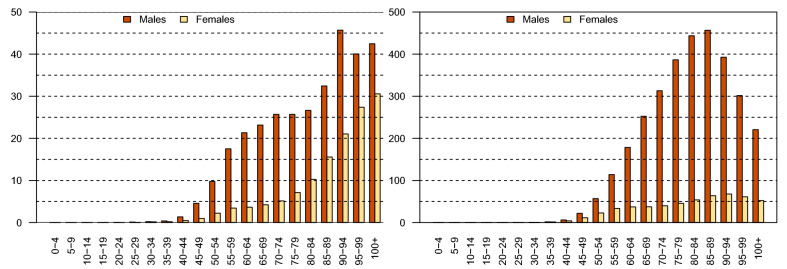
Crude rates divided by age-group and gender for LOCP cancer (left) and lung cancer (right).

As an initial picture of the diseases, crude mortality rates per 100,000 population by age, gender and provinces are provided. Figure 1 shows the crude rates divided by age group and gender for each disease. In both diseases, higher crude rates are observed for males than for females and these differences are more pronounced in lung cancer. Differences are also observed by age groups between diseases. For LOCP cancer, a rate increase is observed from the age of 40 onward. Mortality rates increase rapidly up to age-group 65–69 decelerating up to age-group 85–89. For elderly people, rates are even higher possibly due to a certain rate instability in these groups. Conversely, lung cancer rates present inverse U-shaped pattern in both genders, much more pronounced in males. Rates increase with age reaching a peak in the age-group 85–89. We observe that crude rates for the 0–4 to 35–39 age-groups are very low in both diseases and therefore, we exclude them from the statistical modelling to smooth rates from now on. The next five age groups will be considered: 30–44, 45–59, 60–74, 75–89 and 90+.

**Figure 2:**
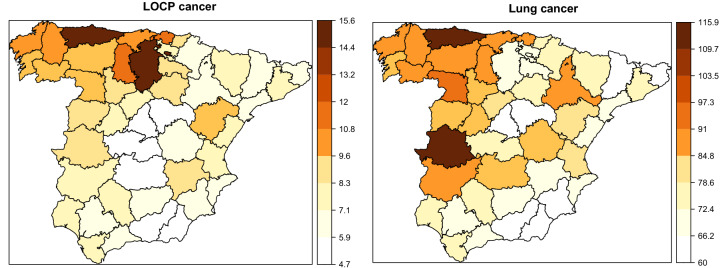
Crude mortality rates for males in continental Spanish provinces for each disease.

**Figure 3:**
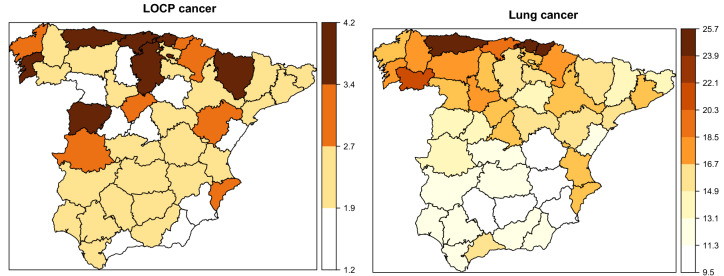
Crude mortality rates for females in continental Spanish provinces for each disease.

To complete the descriptive analysis, Figs. 2 and 3 display the crude rates per 100,000 habitants by province and gender for LOCP and lung cancer. These figures show vast disparities by gender and region. As we observed before, males are more affected than females. With respect to the crude geographical pattern, provinces located in the north-west part of Spain have the highest rates in males. In females, the provinces with the highest rates are located in the north. Therefore, different spatial patterns are observed by gender. These preliminary analyses suggest differences and variability in LOCP and lung cancer mortality rates among genders, age groups and regions. Subsequently, statistical models will be used to smooth rates and reveal the geographical patterns by age group and gender.

## Methods

As mentioned above, in this study we are interested in analyzing the spatial pattern of LOCP cancer mortality rates by age and gender. As the number of LOCP cancer deaths by age, gender and region are scarce, the fit of univariate spatial models does not provide reliable results. Shared component models are used to overcome this issue. These models are appropriate when it is known that the diseases share some common risk factors as in the case of LOCP and lung cancers, where smoking is an example of such risk factors. Shared component models are also very useful to provide both the spatial pattern common to the studied diseases and the disease-specific spatial patterns. A brief description of the models is given below.

Conditional on the rates $r_{digj}$, the number of deaths for each disease, $O_{digj}$, (*d*, $d=1$ for lung cancer and $d=2$ for LOCP cancer), area (*i*, $i=1,\dots ,47$), gender (*g*, $g=1$ male and $g=2$ female) and age-group (*j*, $j=1,\dots ,5$, for age-groups 30-44, 45-59, 60-74, 75-89 and 90+) is assumed to follow a Poisson distribution with mean $\mu _{digj}=n_{digj}r_{digj}$, where $n_{digj}$ is the population at risk. Namely,

1 $$\begin{aligned}&O_{1igj}|r_{1igj}\sim Poisson\left( \mu _{1igj}=n_{1igj}r_{1igj}\right) ; \quad \quad \log \mu _{1igj}=\log n_{1igj}+\log r_{1igj}, \nonumber \\&O_{2igj}|r_{2igj}\sim Poisson\left( \mu _{2igj}=n_{2igj}r_{2igj}\right) ; \quad \quad \log \mu _{2igj}=\log n_{2igj}+\log r_{2igj}. \end{aligned}$$

where the log-rates, $\log r_{digj}$, are now modelled using different proposals. Let us start with the simplest one,

2 $$\begin{aligned}&\log r_{1igj}=\delta \kappa _{i}, \end{aligned}$$

3 $$\begin{aligned}&\log r_{2igj}=\frac{1}{\delta } \kappa _{i}, \end{aligned}$$

where $\delta$ is a scaling parameter and $\kappa _{i}$ represents the shared spatial component. However, this model is not flexible enough because no additional sources of variability are included, other than spatial. As we mentioned above, age groups and gender play an important role in describing LOCP and lung cancers mortality patterns. Then, a set of models including different age, gender and disease interactions are proposed. More precisely, gender-specific shared spatial components were used due to the different spatial patterns observed in Figs. 2 and 3 by gender. In addition, disease-specific effects, age-group effects, and some interactions were also considered. Model fitting and inference were carried out using Bayesian methodology, specifically, integrated nested Laplace approximations (INLA)^13^. One of the advantages of the INLA approach is that it can be implemented in the free software **R** through the R-package *R-INLA*^14^. Finally, to select the best model, the Deviance Information Criterion (DIC)^15^, the Watanabe-Akaike Information Criterion (WAIC)^16^ and the logarithmic score (LS)^17^ were used (see Supplementary Information 2 online for more details). The final selected model is described below.

4 $$\begin{aligned} \log r_{1igj}= & {} \alpha _1+\delta \kappa _{ig}+\eta _{gj}, \end{aligned}$$

5 $$\begin{aligned} \log r_{2igj}= & {} \alpha _2+\frac{1}{\delta } \kappa _{ig}+u_{i}+\eta _{gj}. \end{aligned}$$

Here, $\alpha _1$ and $\alpha _2$ represent the lung and LOCP specific intercept respectively, $\delta$ is a scaling parameter, $\kappa _{ig}$ represents the gender-specific shared spatial component, $u_{i}$ denotes a spatially unstructured random effect and $\eta _{gj}$ are age-gender-specific effects. The following distributions for the effects $\varvec{\alpha }$, $\varvec{\kappa }$, ${\varvec{u}}$ and $\varvec{\eta }$ are assumed

$$\begin{aligned}&\alpha _d \sim N\left( 0,0\text {.}001\right) , \,\, d=1,2\\&p(\mathbf {\kappa }) \propto \exp \left( \frac{-\tau _{\kappa }}{2} \mathbf {\kappa }^{'} {\mathbf {Q}} \mathbf {\kappa } \right) ,\\&{\mathbf {u}}\sim N\left( {\mathbf {0}},\tau _u{\mathbf {I}}_{47}\right) ,\\&\mathbf {\eta }\sim N\left( {\mathbf {0}},\tau _{\eta }\left( {\mathbf {I}}_2\otimes {\mathbf {I}}_7\right) \right) , \end{aligned}$$

where ${\mathbf {Q}}=\left( {\mathbf {I}}_2\otimes {\mathbf {R}}\right)$ is the precision matrix determined by the ${\mathbf {R}}$ spatial neighborhood structure with the *i*th diagonal element equal to the number of neighbours of the *i*th province and for $i\not =j$, $R_{ij}=-1$ if *i* and *j* are neighbours and 0 otherwise. Here two provinces are neighbours if they share a common border. ${\mathbf {I}}_2$, ${\mathbf {I}}_{47}$ and ${\mathbf {I}}_7$ are identity matrices of sizes 2, 47 and 7, respectively. The $\kappa _{ig}$ component gives the common spatial pattern of LOCP and lung cancer mortality for each gender, whereas the spatially unstructured random effect, $u_{i}$ explains the disparities in the spatial distributions of LOCP and lung cancer mortality. This term has been added to the model because of the disparities observed in the spatial patterns among the diseases. Finally the age-gender-specific effect, $\eta _{gj}$, captures the differences between age groups for each gender. As we have seen in Fig. 1 the rates by age-group were different for each gender and this effect tries to capture these disparities.

Prior distributions on the precision parameters (inverse of variance components) are required to fully specify the models. Here, penalized complexity priors (PC-priors) were used^18^. A zero mean normal prior with precision 0.001 was considered for the fixed effects and a loggamma prior distribution with value 10 in both parameters was considered for $\delta$ in log scale. Finally, sum-to-zero constrains are needed to guarantee identifiability of the different model terms^19^. More precisely, $\sum _{i} \kappa _{ig}=0, \,\, g=1, \,\, 2$ for the shared term. These restrictions are imposed in INLA by default.

A sensitivity analysis was also conducted to assess the impact of different sets of hyperpriors on the final rate estimates. Results shown in this paper were obtained using PC-priors but we also considered improper uniform priors on the standard deviations and loggamma priors on the log precisions. Posterior means, medians, and standard deviations for the precision parameters were calculated. PC-priors and improper uniform priors lead to small differences for the age-gender-specific precisions. However, these differences did not affect the final rate estimates (see Supplementary Information 2 online for more details). Results provided by the selected model are given below.

Figures and maps shown in this article were created using the free R software, version 3.5.2 (https://cran.r-project.org/)^20^. All methods were carried out in accordance with relevant guidelines and regulations.

## Results

**Figure 4:**
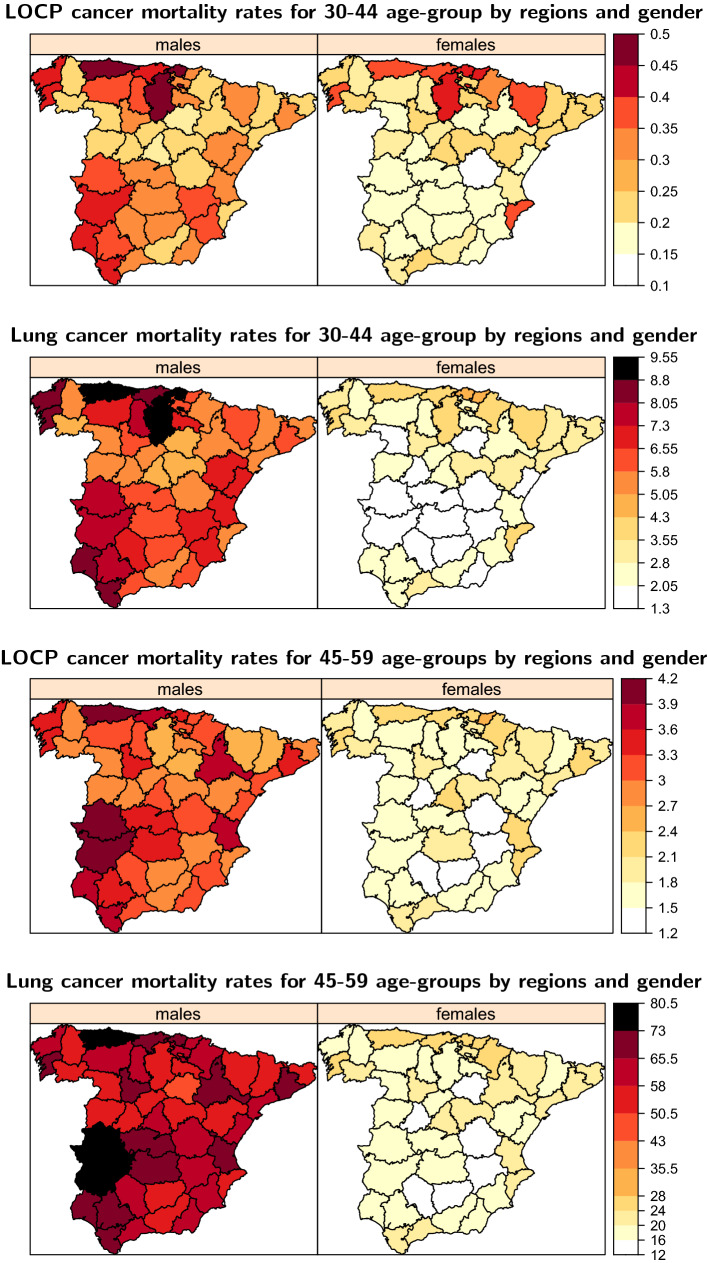
Posterior medians of LOCP and lung cancer gender-specific mortality rates per 100,000 population by regions for the 30–44 and 45–59 age groups.

Once we fitted the selected shared component model explained above, posterior distributions of region, gender and age-specific rates were obtained. For simplicity, we represent in several figures medians of the corresponding posterior distributions (see Fig. 4). Posterior medians of the gender-specific shared spatial component and the spatially unstructured random effect are also given (Figs. 6 and 7 respectively).

### Patterns for young adults

Figure 4 shows posterior medians of mortality rates for LOCP and lung cancers per 100,000 population for the youngest age groups (30–44, 45–59) by gender and province. For 30–44 age group, rates are very low in both diseases. A rate increase is observed for the 45–59 age group, especially for males regarding lung cancer mortality. In the case of LOCP cancer, the rates remain low. Both diseases also present some differences in the geographical pattern between genders. In males, some provinces in the north and south-west of Spain show the highest rates in both age groups in general while in females, the highest rates are located mainly in the north.

### Patterns for older age groups

**Figure 5:**
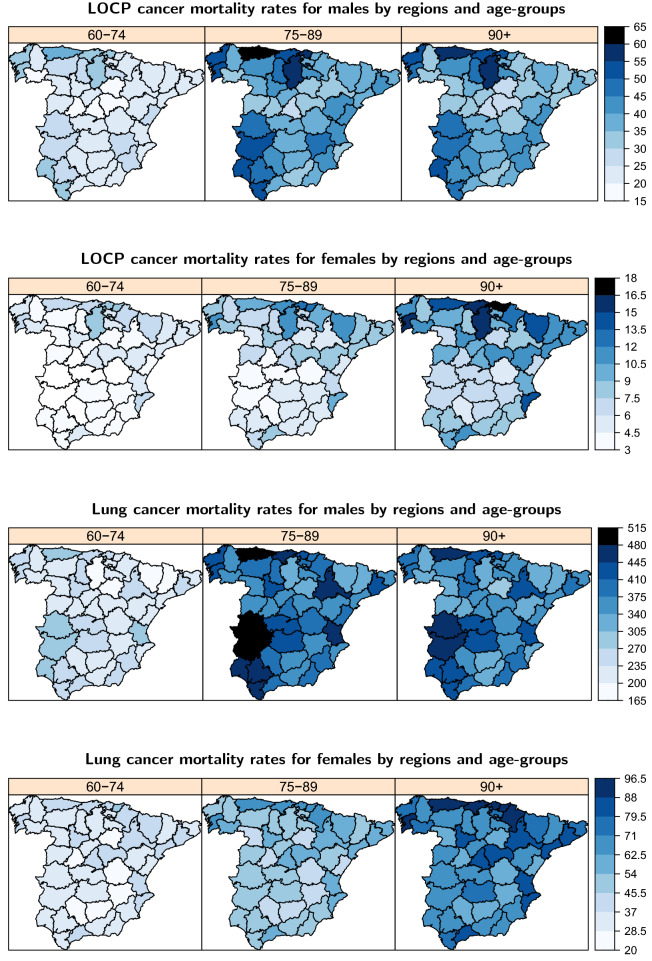
Posterior medians of LOCP and lung cancer gender-specific mortality rates per 100,000 population by regions for the 60–74, 75–89 and 90+ age groups.

Figure 5 provides the posterior medians of mortality rates for LOCP and lung cancers per 100,000 population for 60–74, 75–89 and, 90+ age groups.

Different spatial patterns are observed by age group and gender. Male mortality rates increase from the 60–74 to the 75–89 age-group and then decrease for the 90+ age group, whereas female mortality rates increase with age reaching their maximum in the 90+ age group. For LOCP cancer, Asturias, Burgos, Vizcaya and Cantabria are the provinces with the highest mortality rates in males while Vizcaya, Guipuzcoa, Burgos and Pontevedra present the highest rates in females. Some provinces located south–west Spain also present high male mortality rates. For lung cancer, the highest mortality rates are located in Caceres, Badajoz and Asturias in males, whereas in females, some provinces in the north present the highest rates. This pattern is similar in all age groups.

Table 1 ranks high mortality regions by age-group and gender to better identify the most affected subgroups. Posterior median of mortality rates and their corresponding 95% credible interval are provided. The provinces with the highest rates are the same in the three age-groups within each gender. More precisely, provinces in the north–west and south–west in males and northern provinces in females.

**Table 1:** Posterior medians and 95% credible interval of the ten provinces with highest rates of LOCP cancer by gender for age-groups 60–74, 75–89 and 90+.

Region	60–74		75–89		90+	
	Median	95% Credible interval	Median	95% Credible interval	Median	95% Credible interval
**Males**						
Asturias	35.394	32.155–38.842	60.171	54.663–66.036	56.915	51.280–63.008
Burgos	34.554	29.248–40.523	58.757	49.733–68.909	55.561	46.805–65.499
Vizcaya	33.290	30.165–36.626	56.605	51.288–62.281	53.531	48.109–59.407
Cantabria	32.218	28.003–36.844	54.789	47.618–62.659	51.809	44.772–59.621
La Coruña	31.423	28.389– 34.666	53.423	48.262–58.942	50.531	45.292–56.214
Huelva	31.176	26.318–36.602	53.019	44.754–62.255	50.135	42.117–59.178
Pontevedra	30.400	27.089–33.974	51.689	46.054–57.772	48.885	43.253–55.040
Cadiz	30.299	26.988–33.866	51.527	45.887–57.607	48.728	43.089–54.883
Badajoz	29.501	25.532– 33.847	50.172	43.420–57.563	47.443	40.826–54.765
Caceres	28.662	24.090–33.752	48.744	40.969–57.397	46.092	38.561–54.557
**Females**						
Vizcaya	8.234	7.171–9.418	12.330	10.740–14.101	17.258	14.896–19.926
Guipuzcoa	8.083	6.740–9.630	12.105	10.093–14.421	16.941	14.027–20.330
Burgos	7.511	5.823–9.560	11.248	8.721–14.313	15.741	12.149–20.133
Pontevedra	7.293	6.205–8.530	10.923	9.294–12.774	15.285	12.906–18.021
Cantabria	7.245	5.926–8.787	10.849	8.874–13.155	15.184	12.345–18.532
Huesca	7.074	5.076–9.652	10.593	7.603–14.450	14.825	10.602–20.305
Asturias	6.987	6.060–8.021	10.464	9.078–12.008	14.645	12.596–16.960
Alicante	6.832	5.993–7.768	10.231	8.971–11.638	14.319	12.432–16.453
Navarra	6.321	5.089–7.758	9.452	7.621–11.616	13.228	10.606–16.356
Tarragona	5.408	4.379–6.625	8.099	6.557–9.921	11.334	9.122–13.973

### Common geographical pattern of LOCP and lung cancer mortality

**Figure 6:**
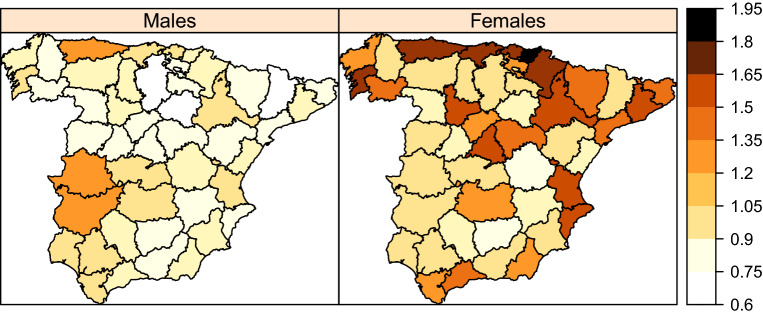
Posterior gender-specific shared spatial patterns for LOCP and lung cancers mortality.

Shared component models provide the common geographical pattern of the two cancers. This can be interpreted as a common risk pattern that both diseases share. To obtain the common geographical pattern of LOCP and lung cancer mortality, the posterior gender-specific shared spatial pattern per 100,000 population, $\exp \left( \varvec{\kappa _{ig}}\right) *100,000$ was calculated. Figure 6 displays the posterior median of the gender-specific shared spatial random effect, indicating the common spatial pattern for both LOCP and lung cancers in each gender. Clearly, the spatial shared effect is stronger (darker) in females. It means that the spatial distribution of lung and LOCP cancer are more similar in females than in males. In males, both diseases present high risks in Asturias, Caceres and Badajoz. In females, the high risk areas are mainly located in the north–east part of Spain. The maps reveal that there are possibly certain risk factors with a spatial pattern that are not affecting both genders equally.

### Particular LOCP cancer spatial pattern differing from lung cancer

**Figure 7:**
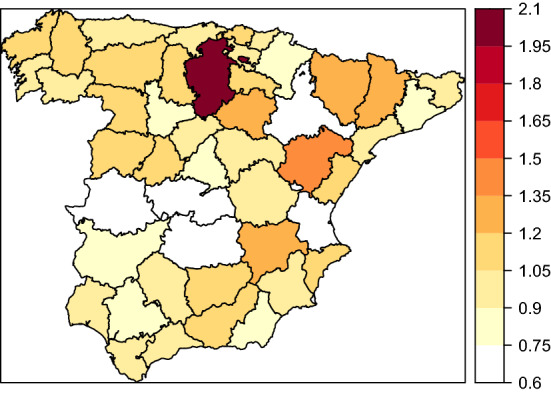
Posterior median of the spatially unstructured random effect pattern for LOCP cancer mortality.

The spatially unstructured random effect has been computed as $\exp \left( {\varvec{u}}\right) *100,000$. Figure 7 displays the posterior median of the spatially unstructured random effect for LOCP cancer, representing the disease and region-specific effects that can not be explained by the shared term. This figure allows to identify LOCP cancer rates singularities and could be useful to identify potential risk factors affecting LOCP cancer mortality but not lung cancer mortality. In this figure, the province of Burgos stands out. This highlights Burgos as a province particularly affected by LOCP cancer mortality.

## Discussion

The purpose of this study was to reveal the geographic distribution of LOCP cancer mortality rates in continental Spain by gender and age groups. We conclude that LOCP cancer mortality rates increase with age, reaching its maximum in the 75–89 and 90+ age groups for males and females respectively, but females present lower mortality rates than males in all age groups. Some geographical hotspots for LOCP cancer mortality rates were found. Namely, Asturias, Burgos, Vizcaya and Cantabria (all located in the north of Spain) are the provinces with the highest mortality rates in males for all age groups. Vizcaya and Burgos are also provinces showing high rates in females in all age groups together with Guipuzcoa.

Differences in LOCP cancer mortality rates by gender are a generalized phenomenon^1^. Several studies indicate that smoking is the most important risk factor in this cancer and differences in smoking consumption have been observed by gender^21–23^. The mass consumerism of tobacco among women was delayed 40–50 years compared to men, and this is mainly attributed to contextual conditioning factors^24^. Among women, smoking was rare before the 1960s in Spain, but tobacco consumption increased from the 1970s onwards, mainly in the generations born after 1940^24,25^. The highest prevalence (49.9%) was found in 1990 among women from the 1970–1979 birth cohort^24^. Smoking prevalence among men, historically higher than in women, has been slowly decreasing since 1980^24,26^ and gender differences are getting closer with time^5,27–29^. This study also found differences by age groups. As mentioned before, mortality climbs as age increases. Smoking prevalence can also be a possible explanation for that. It is known that the years of smoking prevalence and the average number of cigarettes per day boosts the risk of LOCP (and lung cancer)^23,30,31^. Increasing mortality rates with age for both diseases are likely to be a reflection of a longer exposure time to tobacco consumption. We would like to stress that age is an important factor explaining LOCP mortality rates. This is clearly reflected in the data analysis (see Supplementary Information 2 online) as the random effect $\varvec{\eta }$ explains the largest proportion of residual variability.

The geographical hotspots found in this study could be explained by a heterogeneous distribution of LOCP cancer risk factors among Spanish provinces. We conclude that some provinces in the north present high LOCP cancer mortality rates in both genders. As these high rates have been particularly observed in the elderly, it could be the reflection of exposures to risk factors in the past. Historically, these were industrial and mining provinces. Apart from tobacco consumption, exposure to asbestos^32,33^, polycyclic aromatic hydrocarbons^34,35^ or PM$_{2.5}$^36,37^ are highlighted in the literature as other LOCP (and lung cancer) risk factors. Between 1914 and 1918, there was a coal boom in Asturias, increasing the number of coal miners from 18,000 to 39,000, making it the main province for national coal production^38^. Asturias continued to dominate the sector until 1960, when coal suffered a great decline^39^. This aspect could explain some of the results obtained from this study, i.e., the exposure to these risk factors might explain the high rates observed in this province in males. Some studies have reported higher mortality rates for lung cancer in coal mining areas that corroborate what we have seen in our study^40,41^. On the other hand, Vizcaya experienced an industrial growth in 1900 and this development continued throughout the 20th century. The leading industries were iron and steel^42^ in which asbestos-containing products were frequently used in constructions and insulation, for example. In this province, the pollution levels increased meaningfully in this period, also increasing the number of PM$_{2.5}$. The same reason could be used to explain the high rates observed in Cantabria as it has also been closely linked to iron and steel industry growth since 1950^42^. Specifically in the case of males, high mortality rates have been also found in Coruña, Huelva, Pontevedra, Cadiz and Extremadura (Caceres and Badajoz). Huelva and Cadiz are more industrialized regions in the south thanks to the ship building sector^43^. Asbestos had been also very common in the construction of ships and therefore many workers had been exposed to this mineral. The ship building sector is also important in Coruña and Pontevedra. On the other hand, Extremadura is home to more than the 91% of tobacco plantations of Spain and the average of tobacco consumption in Extremadura is above the Spanish average^44^. The case of Burgos is also particularly interesting with high LOCP mortality rates (but low lung cancer rates). Therefore, a specific LOCP cancer risk factor might affect this province. In the epidemiological literature, a poor oral health care, alcohol consumption or a bad diet^21,45,46^ are directly related to LOCP cancer. In Castilla and León, the autonomous region that Burgos belongs to, alcohol consumption is above the Spanish average^47^ but results were not provided by province within this region. This could explain the particular behaviour of Burgos, but further research is needed to corroborate this hypothesis.

To conclude, we would like to highlight the importance of this study as most previous studies in Spain are limited to describing LOCP cancer mortality rates at a local level, using data from local cancer registries, and therefore a general analysis of LOCP cancer mortality patterns in Spain by age groups and gender was missing. We have identified spatial hotspots for LOCP cancer mortality in Spanish provinces for each gender in all age groups. This opens up future research priorities in these areas and suggests the need for further investigation.

## Supplementary Information

Supplementary Figure S1.

Supplementary Information.
