# Supplementary material for: Estimating LOCP cancer mortality rates in small domains in Spain using its relationship with lung cancer
Source: Sci Rep. 2021 Nov 15;11:22273. doi: 10.1038/s41598-021-01765-7 (PMC8593013; doi:10.1038/s41598-021-01765-7)
Supplement: Supplementary file 1 — Supplementary Figure S1. [file 41598_2021_1765_MOESM1_ESM.pdf]

Supplementary Information 1 for:

Estimating LOCP cancer mortality rates in small domains in Spain using its relationship with lung cancer

Garazi Retegui[1,2,3,+], Jaione Etxeberria[1,2,3,+], and María Dolores Ugarte[1,2,3,+,*]

[1]Institute of Health Research (IdiSNA), Pamplona, 31008, Spain
[2]Public University of Navarre, Statistics, Computer Science and Mathematics, Pamplona, 31006, Spain
[3]Institute for Advanced Materials and Mathematics (INAMAT[2]), Public University of Navarre, Pamplona, 31006, Spain
[*]lola@unavarra.es
[+]these authors contributed equally to this work

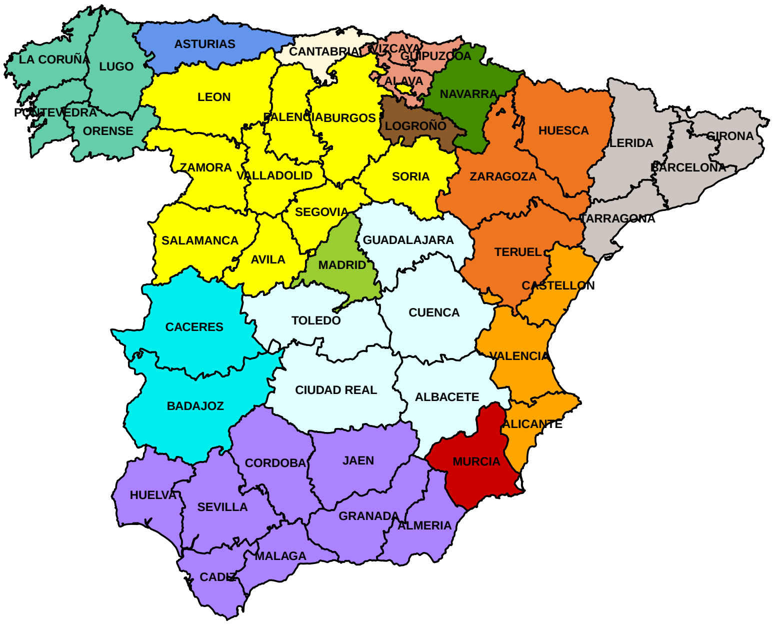

Figure S1: Administrative division of Spain into provinces. Provinces belonging to the same autonomous regions share the same color. This figure was created using the free R software, version 3.5.2. (`https://cran.r-project.org/`)
