# Supplementary material for: Estimating LOCP cancer mortality rates in small domains in Spain using its relationship with lung cancer
Source: Sci Rep. 2021 Nov 15;11:22273. doi: 10.1038/s41598-021-01765-7 (PMC8593013; doi:10.1038/s41598-021-01765-7)
Supplement: Supplementary file 2 — Supplementary Information. [file 41598_2021_1765_MOESM2_ESM.pdf]

Supplementary Information 2 for:

Estimating LOCP cancer mortality rates in small domains in Spain using its relationship with lung cancer

Garazi Retegui[1,2,3,+], Jaione Etxeberria[1,2,3,+], and María Dolores Ugarte[1,2,3,+,*]

[1]Institute of Health Research (IdiSNA), Pamplona, 31008, Spain
[2]Public University of Navarre, Statistics, Computer Science and Mathematics, Pamplona, 31006, Spain
[3]Institute for Advanced Materials and Mathematics (INAMAT^2), Public University of Navarre, Pamplona, 31006, Spain
[*]lola@unavarra.es
[+]these authors contributed equally to this work

1 Proposed models

The most relevant models proposed in the study are given below

$$\begin{aligned}
\text{Model 0:}\quad \log r_{1igj} &= \alpha_1 + \delta\kappa_{ig} + \xi_{gj},\\
\log r_{2igj} &= \alpha_2 + \frac{1}{\delta}\kappa_{ig} + \xi_{gj}.
\end{aligned}$$

$$\begin{aligned}
\text{Model 1:}\quad \log r_{1igj} &= \alpha_1 + \delta\kappa_{ig} + \eta_{gj},\\
\log r_{2igj} &= \alpha_2 + \frac{1}{\delta}\kappa_{ig} + \eta_{gj}.
\end{aligned}$$

$$\begin{aligned}
\text{Model 2:}\quad \log r_{1igj} &= \alpha_1 + \delta\kappa_{ig} + \eta_{gj},\\
\log r_{2igj} &= \alpha_2 + \frac{1}{\delta}\kappa_{ig} + u_i + \eta_{gj}.
\end{aligned}$$

$$\begin{aligned}
\text{Model 3:}\quad \log r_{1igj} &= \alpha_1 + \delta\kappa_{ig} + w_{1i} + \eta_{gj},\\
\log r_{2igj} &= \alpha_2 + \frac{1}{\delta}\kappa_{ig} + w_{2i} + \eta_{gj}.
\end{aligned}$$

Table S1: Model selection criteria for the candidate models.

	DIC	WAIC	LS
Model 0	9625.140	10522.540	5197.493
Model 1	8015.083	8371.011	4196.074
Model 2	7777.089	8275.495	4176.651
Model 3	7798.287	8306.438	4192.215

The final selected model is Model 2.

2 Sensitivity analysis

Table S2: DIC, WAIC and LS values obtained by the selected model with different priors.

	DIC	WAIC	LS
PC-priors	7777.089	8275.495	4176.651
Uniform	7775.170	8273.877	4175.941
Log-gamma	7777.456	8274.818	4176.171

Table S3: Posterior mean, standard deviation, mean and 95% credible interval of the precision parameters and the parameter δ obtained by the model with different prior distributions.

		mean	sd	0.025quant	0.5quant	0.975quant
PC-priors	τ_κ	5.7007	1.1666	3.7974	5.5649	8.3677
	δ	0.6965	0.0187	0.6597	0.6965	0.7332
	τ_u	20.3038	5.4786	11.1172	19.8256	32.4709
	τ_η	0.4689	0.1558	0.2277	0.4484	0.8334
Uniform	τ_κ	5.3973	1.0887	3.5571	5.2963	7.8266
	δ	0.6954	0.0188	0.6590	0.6952	0.7332
	τ_u	19.3908	5.3020	10.9448	18.7361	31.6648
	τ_η	0.2471	0.1240	0.0769	0.2247	0.5514
Log-gamma	τ_κ	5.7547	1.1667	3.6732	5.6950	8.2309
	δ	0.6972	0.0189	0.6591	0.6977	0.7334
	τ_u	21.7747	5.8936	11.8807	21.2647	34.8431
	τ_η	0.3378	0.1474	0.1291	0.3126	0.6979

Regarding the shared spatial component, we would like to emphasize that this effect is absolutely necessary to obtain the geographical distribution of lip, oral cavity, and pharynx (LOCP) cancer mortality rates in Spain by age-group and gender. A univariate gender and age-specific spatial model for LOCP assessed initially didn't provide sensible results due to the small number of cases in these small domains. For that reason, the shared spatial term is indispensable to model LOCP mortality rates. Furthermore, in order to verify that the results of the multivariate model for LOCP cancer are adequate (because the disease with the highest number of cases may have a higher weight on the estimates of the effects), the results of a multivariate spatial model and a univariate spatial model for LOCP (without age-group and gender effects) were compared and, the results were very similar. Finally we note that the gender-age-specific effect) is dominant as it explains the largest proportion of residual variability. This indicates that the age largely explains mortality patterns in both lung and LOCP cancer mortality. This is a common fact in most of the cancers and it has been widely studied in the literature.

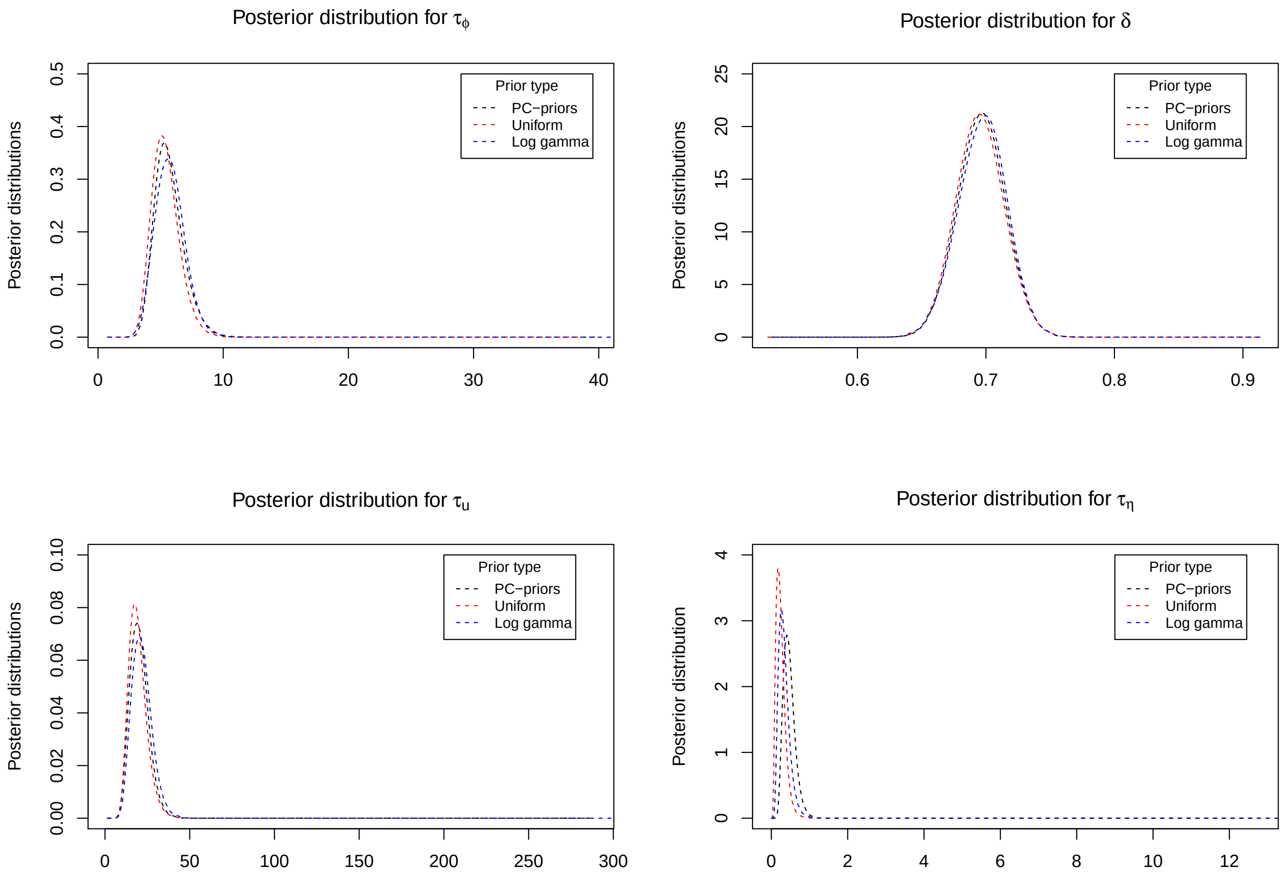

Figure S2: Posterior distributions of the precision parameters of the model obtain by the different priors define for the sensitivity analysis.

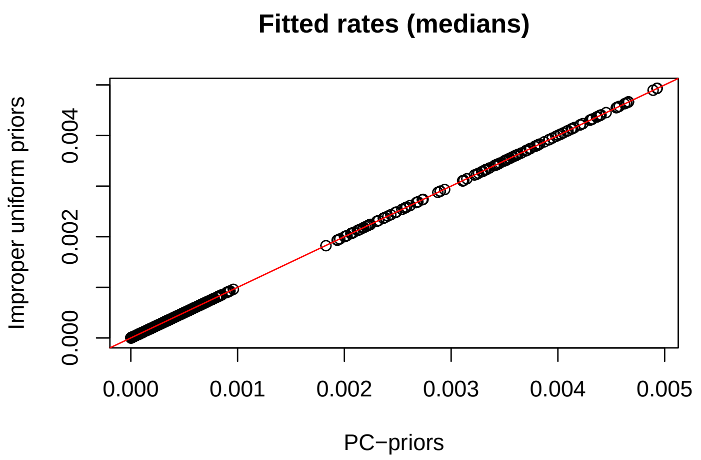

Figure S3: Dispersion plot for the posterior medians of the fitted rates with a PC-prior and a uniform prior.
